# Emergency provider perspectives on facilitators and barriers to home and community services for older adults with serious life limiting illness: A qualitative study

**DOI:** 10.1371/journal.pone.0270961

**Published:** 2022-08-05

**Authors:** Jacob D. Hill, Claire De Forcrand, Allison M. Cuthel, Oluwaseun John Adeyemi, Amanda J. Shallcross, Corita R. Grudzen

**Affiliations:** 1 Ronald O. Perelman Department of Emergency Medicine, New York University Grossman School of Medicine, New York, New York, United States of America; 2 School of Medicine and Public Health, University of Wisconsin, Madison, Wisconsin, United States of America; 3 Department of Population Health, New York University Grossman School of Medicine, New York, New York, United States of America; University of Auckland, NEW ZEALAND

## Abstract

**Background:**

Older adults account for a large proportion of emergency department visits, but those with serious life-limiting illness may benefit most from referral to home and community services instead of hospitalization. We aim to document emergency provider perspectives on facilitators and barriers to accessing home and community services for older adults with serious life-limiting illness.

**Methods:**

We conducted interviewer-administered semi-structured interviews with emergency providers from health systems across the United States to obtain provider perspectives on facilitators and barriers to accessing home and community services. We completed qualitative thematic analysis using an iterative process to develop themes and subthemes to summarize provider responses.

**Results:**

We interviewed 8 emergency nurses and 10 emergency physicians across 11 health systems. Emergency providers were familiar with local home and community services. Facilitators to accessing these services include care management and social workers. Barriers include services that are not accessible full-time to receive referrals, insurance/payment, and the busy nature of the emergency department. The most helpful reported services were hospice, physical therapy, occupational therapy, and visiting nursing services. Home-based palliative care and full-time emergency department-based care management and social work were the services most desired by providers. Providers expressed support for improving access to home and community services in the hopes of decreasing unnecessary emergency visits and inpatient admissions, and to provide patients with greater options for supportive care.

**Conclusion:**

Obtaining the perspective of emergency providers highlights important considerations to accessing HCS for older-adults with serious life-limiting illness from the emergency department. This study provides foundational information for futures studies and initiatives for improving access to home and community services directly from the emergency department.

## Introduction

The emergency department (ED) is at the crossroads of inpatient and ambulatory care. The decisions made by emergency providers can significantly impact the care trajectory of patients, especially older adults with serious life-limiting illness [1]. Historically, the culture of the ED environment has been to reflexively provide invasive life-sustaining procedures [2]. However, these intensive therapies do not always result in better health, longer life, or consider patient preferences at the end of life [3, 4].

Interest in ED-based palliative care is increasing, and ED-palliative care partnerships have been shown to improve quality of life and potentially reduce length of hospital stay and increase hospice utilization [5]. With the rapidly growing older adult population [6], and high ED-utilization by older adults [7, 8], it essential that emergency providers are aware of, and have access to, home and community services (HCS). Access to HCS can support care trajectories that match the preference and clinical needs of older adults with serious life-limiting illness.

Numerous factors have been associated with reduced access to HCS for older adults with serious illness. These include disease type, lack of provider knowledge on expected disease progression, lack of prognosis acceptance by patients, rural vs urban location, poor communication across healthcare teams, general curative focus in medical care, as well as racial, gender, sexual orientation, and socioeconomic disparities [9–14]. Less is known about facilitators and barriers to accessing HCS from the ED, and specifically the perspective of emergency providers regarding the barriers and facilitators to linking patients to HCS. Since the ED is a critical juncture in the care of older adults, it is important to identify ED-specific variables that influence access to HCS. Identifying these facilitators and barriers can lead to interventions to improve care transitions for older adults with serious life-limiting illness.

This study aims to document facilitators and barriers to accessing HCS from the perspective of emergency physicians and nurses through the use of qualitative interviews. We collected information on 1) familiarity with local HCS, 2) helpful and desired HCS services, 3) provider-identified barriers and facilitators to HCS, and 4) procedures involved when referring to HCS. This study is a sub-project of a national multi-site, pragmatic, cluster-randomized stepped-wedge trial titled *Primary Palliative Care for Emergency Medicine* (PRIM-ER) [15, 16].

## Methods

This study was approved by the New York University Institutional Review Board (ID# i18-00607_MOD10). Verbal consent was provided by all participants.

### Study design

This study used interviewer-administered semi-structured interviews with traditional grounded theory approach and iterative thematic analysis to observe the perceived barriers and facilitators to accessing HCS for older adults with serious life limiting illness [17]. The traditional grounded theory approach emphasizes the discovery of emerging patterns from the data and it differs from the constructivist grounded theory approach, in which the researcher co-constructs the theory along with the participants [18, 19]. We engaged in an iterative process between data collection, analysis, and concepts generation and identified emerging patterns from the data until the concepts were bounded and the body of data was deemed saturated.

### Study participants and recruitment

We used expert sampling, a type of purposive sampling method, to identify key informants from the PRIM-ER site leadership who could provide information based on their knowledge, experience, and expertise [20, 21]. Participants were physicians and nurses from a variety of EDs across the United States (US) who were actively practicing medicine at their respective EDs during the time of their interviews.

### Study procedures

Interviews were conducted via ZOOM teleconferencing services and via phone. The interviews were conducted by the lead author, JDH, using a pre-scripted interview guide (see S1 File). The interviewer has previous training and experience conducting interviews, as well as experience with qualitative analysis. The interview guide was developed by authors JDH, AC, and CRG prior to initiation of the interviews and pilot tested with an emergency medicine physician to ensure clarity and relevance. Interviewees were sent the interview questions in advance of their scheduled interview. All interviewees were educated on the topic and goals of the study and were introduced to the interviewer prior to study initiation.

The interview questions were developed to better understand the provider’s perspectives to facilitators and barriers to HCS available at their respective ED. For this study, we defined HCS as services such as hospice, nursing homes, home health, home care, rehabilitation, physical therapy, occupational therapy, skilled nursing care, etc. The interviewees were reminded prior to starting the interview, and throughout the interview, that we were interested in discussing services that are available outside of their hospital system, such as services provided in a patient’s home or private/community-based organizations for care of older adults with serious life-limiting illness. The interviews were audio-recorded, and notes were documented throughout each interview. Debriefing sessions occurred among the research team after every 3^rd^ interview to review interviewee responses, adjust the interview guide as needed for clarity and relevance, and assess for data saturation. All interviews occurred between December 2019 –March 2020. All interviews took 45 minutes or less. After 18 interviews, we achieved data saturation, defined as the point when no novel information emerged from the data.

### Analysis

We analyzed the notes of the interviews to assess for emerging themes. We transcribed verbatim the recorded audio files of each interview using Landmark Associates Inc. transcription services. The intent of transcribing the recorded audio was to use the participants quotations to develop the themes. Two members of the research team developed the initial codebook, and the codebook was revised at every debriefing session. The research team compared and reviewed codes to achieve consensus on the themes that emerged from the data.

We performed a thematic analysis using an inductive approach [22]. First, we pre-coded the notes of the interviews, generating initial codes. Thereafter, we analyzed the transcripts for additional inductive codes that were missed in the interview notes. All transcripts were independently double coded by members of the research team. We compared codes across all transcripts during the final analysis phase and condensed codes into meaningful themes. Themes were generated with the aid of Dedoose qualitative analysis software version 8.3.43 [23]. Disagreements on coding among independent coders were reconciled via further review and discussion of the transcript until consensus was achieved. We completed cross-investigator and cross-interview analyses. We presented the results as quotations of the study participants, while removing the interviewer’s filler words for clarity and brevity.

### Methodological rigor

To enhance the credibility of this study, we reported how the participants were guided by specific questions in the interview guide. We created a codebook, and we iteratively reviewed the codebook as new information emerged from the data. All members of the research team agreed on the codes and the themes in the final codebook. Furthermore, we used the participants’ quotes to create the themes. In ensuring dependability, we have provided the questions in the interview guide and the procedure of the coding process. To demonstrate the transferability of this study, we provided contextual descriptions of the study participants and the analytical process. The lead author maintains the audit trail of all the interview notes, debriefing sessions, and analytical decisions for the purpose of auditability.

## Results

We completed 18 interviews with 8 emergency nurses and 10 emergency physicians from 11 different health systems throughout the US. Two providers did not complete an interview due to either no response to the initial email, or they needed to cancel due to the busy nature of their ED from the COVID-19 pandemic. The interviewees were a majority White (n = 13) and female (n = 15). Demographic information on interviewees can be found in Table 1. The geographic distribution of our interviewees includes 9 states; Delaware (n = 1), Florida (n = 2), Louisiana (n = 1), Massachusetts (n = 2), Michigan (n = 2), New York (n = 3), Ohio (n = 2), Pennsylvania (n = 3), and Texas (n = 2).

**Table 1:** Interviewee demographics.

Category	Sub-Category	Participants (n = 18)
**Age^a^**		
	20–29	1
	30–39	9
	40–49	4
	50–59	1
	60–69	1
**Gender^b^**		
	Female	15
	Male	2
**Race^b^**		
	Black	2
	Asian	2
	White	13
**Provider Type**		
	Nurse	8
	Physician	10
**Years in Practice^b^**		
	<5	1
	5–10	8
	11–15	5
	>15	3
**Location**		
	Allegheny Health Network	2
	Baystate Health	2
	Christiana Care Health System	1
	Henry Ford Health System	2
	Ichan School of Medicine at Mount Sinai	2
	New York University Langone Health	1
	Ochsner Health System	1
	The Ohio State University Wexner Medical Center	2
	University of Florida Health System	2
	University of Pennsylvania Health System	1
	University of Texas Health Network- MD Anderson	2

a. Age information unavailable for 2 interviewees

b. Gender, race, and years in practice information unavailable for 1 interviewee

Our results yielded 5 major themes; 1) provider familiarity with local HCS, 2) provider opinions on helpful and desired HCS, 3) benefits HCS provide to patients, 4) provider perspectives on barriers and facilitators to accessing HCS, and 5) staff and procedures involved when referring patients to HCS. During our iterative debriefing sessions following every 3^rd^ interview, we compared themes that emerged from nurses versus physicians to compare differences, and no significant differences were seen across any of the themes obtained. Therefore, the following results summarize all provider responses collectively.

### Provider familiarity with local HCS

Providers were familiar with local HCS and able to provide a general list of services as well as names of specialty services, such as programs or organizations who provide care specific for patients with dementia.

*“I feel like we have the usual home health services that are available*. *There’s regular home health, hospice, nursing homes, and some adult daycare programs. There are like 50 hospices, quite a huge amount, given that it’s a very small city. Other things that we have down here is an extended acute care hospital system. These are long-term acute care hospitals. I think they have the most of them down here, I think has the highest concentration of them.”—Participant 16, Physician*

The most frequently mentioned HCS include hospice, home aides, and nursing homes. Additional services mentioned include home hospice, physical therapy, caregiver respite, skilled nursing care, hospital at home, occupational therapy, home-based palliative care, transportation services, adult day care, counseling, general education, insurance counseling, home medical equipment, and sub-acute rehabilitation. Additionally, interviewees provided details on specialty programs for specific populations their health systems serve such as geriatric specific mixed-service organizations, religious groups (ex. Jewish community services) and/or specific disease cohorts (ex. patients with dementia).

*“We have a very large Jewish community here, and they have their own Jewish living facilities as well as home care agencies.”- Participant 6*, *Nurse*

*“There’s home hospice resources, and then there’s a program called PACE, Program of All-Inclusive Care for the Elderly. It’s a government grant-funded program for low-income senior citizens. It has the potential for really comprehensive care. They offer all kind of geriatric care, including their own hospice program within it”- Participant 2*, *Physician*

### Provider opinions on helpful and desired HCS

Providers found hospice, physical therapy, occupational therapy, and visiting nursing services to be the most helpful HCS to support their patients. The most desired services that providers wish they had access to, or improved access to, include home-based palliative care, full time (24/7) social work/case management for improved referral workflow, greater caregiver support, more general education on HCS, and greater long-term follow-up with patients. All providers reported they feel it is important for emergency providers to be aware of HCS and have systems in place to expedite referrals to these services.

*“If we know the community in which our patients are coming from and the resources that are available and the support that can be accessible for those patients, I think overall the care of the patient can be strengthened, and it can be set up for greater success for whatever the outcome we anticipate being for the specific patient. I think that’s a responsibility of all professions to know what resources are available.”- Participant 12*, *Physician*

### Benefits HCS provide to patients

Interviewees believed HCS play an important role in helping patients attain care goals. For example, one provider said;

*“I feel like it’s [HCS] important to not only take care of their medical needs, but to take care of their holistic needs too. Help them do what they want to do at the end of life and not be contained in a hospital. Help them see who they want to see and do what they want to do and be comfortable, not only medically, but also spiritually, and have them tie up all their loose ends. I think that home health aides and nurses can really help with that that aspect too.”—Participant 14*, *Nurse*

Numerous benefits of HCS for older adults with serious life-limiting illness were identified by our interviewees. The most frequently mentioned benefits include avoiding the ED and inpatient admission, symptom management, and caregiver/family support. Additional benefits identified include supporting activities of daily living/home support, personalized care focusing on cultural and/or religious needs, improved medication management, a more holistic approach to care including spiritual, functional, and emotional support, as well as improved support and education on the process of dying.

*“I think end-of-life needs are very complex. Medical nursing care, cultural community and religious aspects can be better addressed in the home or community setting. Those components to care are more at the forefront and can be introduced in a more respectable honor fashion. Community access, information and care—the more heavily that is involved in that community setting, then hopefully that would impact patients having to come into the hospital*.

*There’s so many challenges, obstacles, and higher risk situations that happen for these patients who are potentially at end of life when they do come into the hospital, and it can make it a less personalized experience for that person in that time. If home and community services were more readily accessible and a little bit more attention or emphasis was placed in that environment, I think it would add to the holistic care of the patient.”- Participant 18*, *Nurse*

### Barriers and facilitators to accessing HCS

The most frequently mentioned barriers to accessing HCS include services that are not accessible full-time (24/7) to receive patient referrals, insurance/payment barriers, and the busy nature of the ED. Additional barriers mentioned include inadequate staffing to support HCS referrals, patients refusing services, the location/distance of patients when trying to establish HCS referrals, provider reluctance to refer to services, the requirement to obtain permission from other managing providers (ex. oncologist) prior to a referral, provider preferences to admit patients to the hospital, and having a smaller ED with limited resources.

*“When we have so many inpatients that we’re holding—we’re the biggest inpatient unit in the hospital, and then we have ER patients coming in on top of that, I feel like sometimes we don’t always have the time to address those needs.”- Participant 14*, *Nurse*

Interviewees almost unanimously cited case management and social workers as being the greatest facilitators for linking patients to community services.

### Staff and procedures for referring patients to HCS

Most interviewees mentioned contacting case management and social workers as their primary means of facilitating a patient’s referral to HCS. For instance, one participant mentioned,

*“We do have social work in our ER 24/7. They take the main responsibility for offering these services to the families, to the patients—how to get in touch with resources, things like that.” -Participant 15*, *Nurse*

Additional processes for referral that were frequently mentioned are an assessment of the patient’s physical condition, a referral placed through the electronic medical record, having a discussion with the patient/family, and having the patient meet with a HCS representative. When discussing the key staff members in the referral process, ED-based case management and social workers were most frequently mentioned, followed by general hospital-based case management and social workers.

## Discussion

Our provider interviews highlight several important points regarding familiarity with, and access to, HCS among emergency providers from a variety of locations across the US. Previous studies have examined accessing community resources from the patient and community organization perspective [24]. Samuels-Kalow et al. found multimodal social risk screening tools in the ED and accurate databases for local health services are strategies that may improve access to community resources [24]. This supports our findings, as multiple providers mentioned they desired greater education and resources pertaining to locally available services. However, our results add novel data on the provider perspective to accessing HCS specifically from the ED environment, and identify numerous targets for future interventions aimed at improving access to HCS from the ED.

Despite expressing desire for greater resources on local HCS, our interviewees were familiar with local services, and this did not appear as a barrier to accessing care. Providers identified a wide variety of HCS, as well as specialty end-of-life services and those for specific subpopulations, such as religiously affiliated services. Providers appeared comfortable with their knowledge of HCS and the local population they connect to HCS. There was a desire to remain up to date on local services, so regular education sessions on new or changing HCS may help providers further expand their knowledge. Our data supports previous literature showing ED palliative care collaboration and education for emergency providers is important to improve care [25], and greater HCS education for providers may help reduce the provider reluctance to refer to HCS that was mentioned by the interviewees. Overall, provider familiarity with HCS was confirmed in the interviews, which allows providers to discuss clinically appropriate HCS prior to referring to a care manager or social worker to coordinate referrals.

Providers identified hospice as the most readily available and helpful service for patients. Home-based palliative care and home-based hospice were not perceived as readily available, but highly desired and should be considered when developing interventions to improve end-of-life care for older adults. There was a general desire for greater access to services delivered directly in home versus a community setting, which may reflect patients’ desire to, “age in place,” and die at home [26–28].

Our study identified numerous barriers to accessing HCS. ED workflow management, staffing, and HCS hours of operation were central themes to barriers of accessing services. The busy nature of the ED was repeatedly mentioned as a barrier, and ED overcrowding is an issue worldwide that was magnified by the COVID-19 pandemic [29, 30]. Several providers conveyed the inability to consider or facilitate referrals to HCS because of high ED volume. Providers expressed concern that instead of a busy ED increasing referrals out to HCS to reduce patient volume, overburdened EDs decrease HCS care coordination and encourages more admission to inpatient care. Providers identified greater utilization of HCS as a potential answer to ED overcrowding. HCS may not only reduce ED volume, but also provide better care to older adults in need of end-of-life services by providing more personalized care.

The providers mentioned a lack of availability of case managers and social workers is another important theme that can reduce access to HCS, and one previously identify as a limitation to accessing services [2]. Providers highlighted the various strengths and limitations in staffing models for case managers and social workers. The least assessable model being case managers and social workers that have offices physically located outside of the ED (elsewhere in the hospital) who are not available 24/7. In contrast, case managers and social workers that are physically located in the ED and/or are available 24/7 were identified as facilitators to accessing HCS. This raises concern for inequitable access to services for smaller EDs with less flexibility with staffing. Providers reported smaller EDs have greater challenges with managing high patient volume, greater staffing challenges, and fewer available resources in general. In addition, EDs in rural locations are also at risk for inequitable access to services due to staffing limitations, which has been identified previously as a concern for quality end-of-life care [14, 31]. Personnel management to improve case managers and social workers representation in the ED should be a foundation for developing interventions to improve access to care, and previous studies indicate efforts are needed to improve ED workflow for safer and more efficient patient care [32].

The additional barriers mentioned, including insurance/payment limitations, difficulty connecting patients to HCS for those who live far away from the ED, or the need to obtain permission from other providers before an HCS referral is placed, highlights the highly complex and multifaceted issues that challenge current practices in end-of-life care in the US. Improving care will require large scale changes in healthcare policy and reform and improved coordination among payers, providers, and administrators. However, interventions that focus on more easily modifiable factors to improve care, such as ED staffing models, education, and workflow, can begin to provide relief to millions of older adults nearing the end of life who present to the ED.

### Limitations

While the geographic spread of our participants was varied, there was no provider representation from the western half of the US due to the timing of the interviews and the COVID-19 pandemic. In addition, most of our interviewees were white females, which limits obtaining perspectives from male providers or providers from minority groups. Interviewees were also providers who served as site leaders for the PRIM-ER research study, which is a national palliative-care study, and thus are highly engaged in this topic area and may not represent the general providers knowledge on this topic. Their advanced knowledge and interest in palliative care due to their involvement in the PRIM-ER study may bias their answers towards a greater familiarity of local HCS compared to other emergency providers.

## Conclusion

Our emergency provider interviewees expressed strong support for improving access to home and community services. Improved ED workflow and staffing, as well as coordinating hours of availability for HCS were core recommendations by providers for improving ED access to HCS. Improved access may decrease unnecessary ED visits and inpatient admissions and provide patients with greater care quality and options at the end of life. Locally available HCS were familiar to our emergency provider interviewees, but most expressed a desire for greater HCS education. Our study supports the need for continued efforts in clinical education, workflows, and research on HCS to develop new interventions for an improved ED experience for older adults with serious life-limiting illness.

## Supporting information

S1 File Qualitative interview questions. (DOCX)
