# Supplementary material for: Emergency provider perspectives on facilitators and barriers to home and community services for older adults with serious life limiting illness: A qualitative study
Source: PLoS One. 2022 Aug 5;17(8):e0270961. doi: 10.1371/journal.pone.0270961 (PMC9355176; doi:10.1371/journal.pone.0270961)
Supplement: S1 File — (DOCX) [file pone.0270961.s001.docx]

**Supplemental File 1: Qualitative Interview Questions**

1. Let’s begin by discussing home and community resources for older adults with serious life-limiting illness that are available around *[participant’s healthcare system]*. Can you describe any home and community health services available in your area?
   1. Can you explain which of these services, if any, you find to be particularly helpful for older adult patients with serious life-limiting illness, and why?
   2. Which services, if any, do you wish were more readily available for this patient population, and why?

1. How might home and community health services support a patient’s end-of-life needs?
   1. Do you think it is important for emergency providers to be aware of these services? Please explain.
   2. Do you think it is important for EDs to have a system in place to refer patients to these services? Please explain.

1. What specific process(es) are used to provide, or refer for, these services?
   1. Can you please discuss how these services are similar, or different, at the other PRIM-ER sites within your healthcare network, which include *[name of other PRIM-ER sites if applicable]*?

1. What barriers to do you think exist, or you experience, when trying to connect older adults with serious life-limiting illness in the ED to home and community health services?
   1. What facilitators are in place that help when trying to connect older adult patients with serious life-limiting illness in the ED to home and community health services?

1. Can you provide an example how community health services were leveraged through the PRIM-ER intervention?
   1. Reflecting on the intervention, what could we have been done differently to better integrate and align the PRIM-ER goals to the services available?

1. Is there anything else we have not talked about that you would like to add?
